# Endometriosis of the Vermiform Appendix within a Hernia Sac Infiltrating the Pubic Bone

**DOI:** 10.1155/2015/270206

**Published:** 2015-01-19

**Authors:** Damian Ziaja, Tomasz Bolkowski, Krzysztof Januszewski, Wioletta Skrzypulec-Plinta, Jerzy Chudek, Krzysztof Ziaja

**Affiliations:** ^1^Department of General and Vascular Surgery, Medical Faculty in Katowice, Medical University of Silesia in Katowice, Ziolowa Street 45-47, 40-635 Katowice, Poland; ^2^Department of Physiotherapy, Faculty of Health Sciences, Medical University of Silesia in Katowice, Medykow Street 12, 40-752 Katowice, Poland; ^3^Department of Pathology, Silesian Medical Center, Silesian Medical University in Katowice, Ziolowa Street 45-47, 40-635 Katowice, Poland; ^4^Department of Women's Disease Control and Prevention, Faculty of Health Sciences, Medical University of Silesia in Katowice, Medykow Street 12, 40-752 Katowice, Poland; ^5^Department of Pathophysiology, Medical Faculty in Katowice, Medical University of Silesia in Katowice, Medykow Street 18, 40-752 Katowice, Poland

## Abstract

*Purpose*. Appendicular endometriosis mimicking appendicitis is a rare finding. Inguinal tumor in the course of appendicular endometriosis located within an inguinal hernia sac and infiltrating the periosteum of the pubic bone has not yet been described. *Case Report*. This paper describes a case of a rapidly enlarging, solid, unmovable, very painful upon palpation inguinal tumor, in a 36-year-old nulliparous woman. During surgery, a hard (approximately 4 cm in diameter) tumor infiltrating the periosteum of the right pubic bone and continuous with the inguinal hernia sac was dissected. The distal segment of the vermiform appendix was an element of the dissected tumor. Histological examination revealed endometriosis of the distal vermiform appendix. After 6 months of hormone treatment, she was referred for reoperation due to tumor recurrence. Once again histological examination of the resected tissue revealed endometriosis. There was no further recurrence of the disease with goserelin therapy. In addition to the case report, we present a review of the literature about endometriosis involving the vermiform appendix and the inguinal canal (Amyand's hernia). *Conclusion*. This case expands the list of differential diagnoses of nodules found in the inguinal region of women.

## 1. Introduction

Appendicitis is the most common surgical disease. The intraoperative finding of an inflamed appendix in the inguinal hernia sac is a rare pathology. It was first described in 1736 by Amyand, who removed a ruptured appendix located within the inguinal hernia of an 11-year-old boy suffering from a spontaneous appendico-cutaneous fistula [1]. Since that time, the pathology has been described by numerous authors [1–3].

Appendicular endometriosis mimicking appendicitis is another rare finding [4–9]. Arévalo Suárez and Cerrillo Sánchez described a series of four cases of such localized endometriosis [9]. However, appendicular endometriosis located within the inguinal hernia sac and infiltrating the periosteum of the pubic bone has not yet been described.

## 2. Case Report

A 36-year-old woman with regular menstruation cycles, presented with pain in the right lower abdomen under observation at our surgical outpatient clinic 6 months previously, returned to the clinic complaining of a painful mass localized on the right pubic bone. She had noticed the mass 3 days prior. An open right inguinal canal had previously been diagnosed by passing a fingertip during a painless palpation, but sac formation was not detected during the cough test.

The patient was urgently referred to the inpatient surgery department. The inguinal mass was a solid, unmovable, very painful upon palpation inguinal tumor, which had a diameter of approximately 4 cm, and was located over the right pubic bone. The skin above the tumor was warm. The patient reported regular menses and was menstruating on admission. The laboratory workup revealed only a borderline elevated white blood cell count (11.9 × 10^3^/*μ*L).

**Figure 1:**
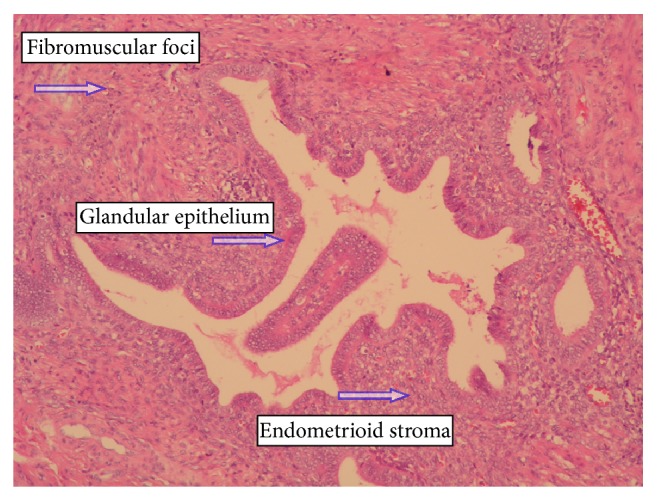
Appendix wall focusing on endometriosis inside. Epithelial and stromal components are present. H-E staining. Magnification 180x.

**Figure 2 fig2:**
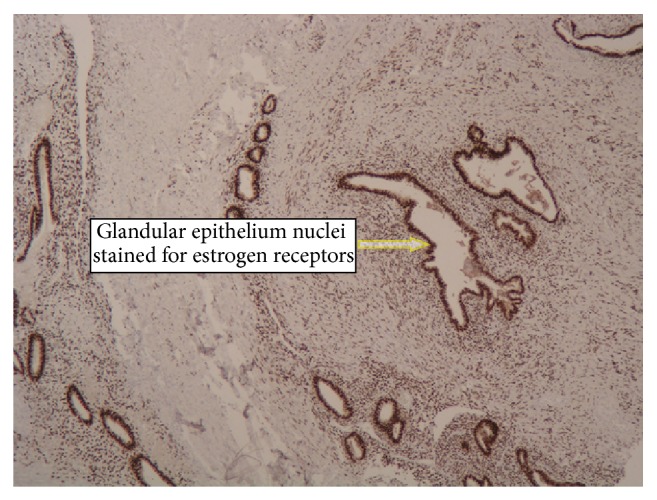
Darkly brown stained nuclei of epithelial cells of endometriosis foci show positive reaction for estrogen receptors. IHC staining for Rer. Magnification 120x.

**Figure 3:**
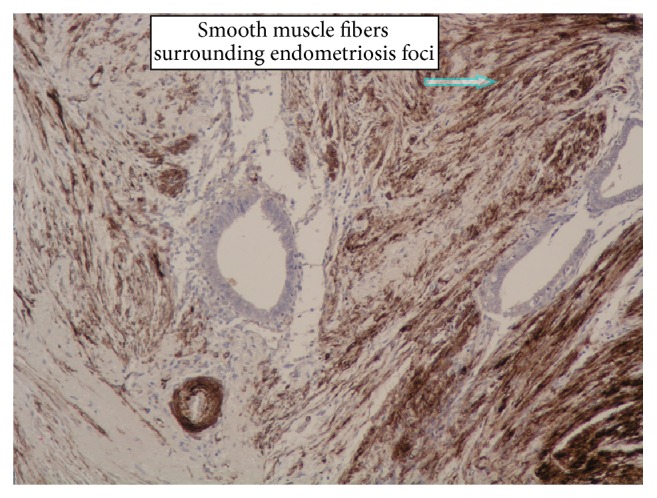
Darkly brown stained smooth muscle fibers of the appendix's muscularis propria show positive reaction form smooth muscle actin surrounding endometriosis foci. IHC staining for SMA. Magnification 250x.

Surgery was performed under general anesthesia. A solid tumor (approximately 4 cm in diameter) infiltrating the periosteum of the right pubic bone in continuity with the inguinal hernia sac was dissected. The distal segment of the vermiform appendix (10 cm long) was an element of the dissected tumor. The vermiform appendix was removed, and the stump of the cecum was sutured after disinfection with iodine and returned to the peritoneal cavity. Histological examination revealed endometriosis of the distal vermiform appendix (Figures 1 [Fig fig2]–3).

The patient underwent further treatment under the supervision of the Gynecology Outpatient Clinic. After 6 months of hormone treatment with a 2 mg daily dose of dienogest (a nortestosterone derivative with antiandrogen action), which did not influence her menstrual cycle, she was referred for reoperation due to tumor recurrence.

On admission, another 4 cm unmovable tumor, which was painless on palpation, was discovered. No other symptoms were reported. The right inguinal canal was closed and the tumor was excised completely (with approximately 1 cm margins) under spinal anesthesia. Histological examination revealed endometriosis. The postoperative course was complicated by local infection, which subsided during an empiric antibiotic therapy.

After surgery, the patient again underwent treatment with goserelin (synthetic analogue of gonadotropin-releasing hormone) for 9 months, this time followed by a monophasic contraceptive (drospirenone + ethinyl estradiol) under the supervision of the Gynecologic Outpatient Clinic. No recurrence of the disease has been reported.

## 3. Discussion

A case of a tumor of the pubic bone with appendicular endometriosis is unique in surgical and gynecological literature. The tumor infiltrating the superior pubic rami probably developed slowly and was asymptomatic for considerable time. The sudden appearance of symptoms was not connected to trauma or gynecological, bone, or joint disease. Interestingly, the patient did not report menstrual pain, which is typical for endometriosis. In our opinion, the development of pain was related to the infiltration of periosteum in the late stage of the disease. It remains a matter of speculation whether the endometriosis initially involved the peritoneum of the inguinal hernial sac and then involved the vermiform appendix, or vice versa.

The presence of the vermiform appendix in the inguinal hernia sac accounts for 1% of all cases of inguinal hernia, whereas acute appendicitis at this location accounts for approximately 0.1%–0.13% of cases [10–13].

Appendicular endometriosis is a rare pathology, usually diagnosed during operations for acute appendicitis [14, 15]. This pathology has also been reported in patients with an incarcerated inguinal hernia [16, 17]. Additionally, endometriosis of the inguinal canal has been described in several case reports as a painful nodule with menstrual variability [17] or as a painless inguinal mass similar to a recurrent hernia [18]. In addition, numerous cases of abdominal wall endometriosis, usually developing in postoperative scaring, have been reported [19, 20].

The diagnosis of appendicular endometriosis infiltrating pubic bone is difficult. Only surgical exploration and histological examination allow the proper diagnosis in such cases.

## 4. Conclusion

This case expands the list of differential diagnoses of nodules found in the inguinal region of women.
